# Micronucleus Frequency Test in Epithelial Cells Desquamated From the Oral Mucosa of Patients With Fanconi Anemia

**DOI:** 10.1111/jop.70166

**Published:** 2026-07-10

**Authors:** Bárbara Soldatelli Ballardin, Pâmela Olivia de Moura, Pedro Leonardo Czmola de Lima, Juliana Lucena Schussel, Cassius Carvalho Torres‐Pereira

**Affiliations:** ^1^ Department of Stomatology Federal University of Paraná Paraná Brazil

**Keywords:** exfoliative cytology, Fanconi anemia, micronuclei test, oral cancer, oral potentially malignant disorders

## Abstract

**Background:**

Fanconi anemia (FA) is a rare genetic disorder predisposing patients to oral squamous cell carcinoma (OSCC). Hematopoietic stem cell transplantation (HSCT) corrects bone marrow failure but may increase the risk of malignancies. Exfoliative cytology and the micronucleus (MN) test provide noninvasive assessment of genomic instability.

**Objective:**

To compare relative frequency of micronuclei (RFMN) in intact mucosa and OPMD lesions in FA patients, and to evaluate its correlation with time post‐HSCT.

**Methods:**

Forty‐six FA patients were included: Group 1 (FA without HSCT, *n* = 14), Group 2 (FA post‐HSCT without OPMD, *n* = 13), and Group 3 (FA post‐HSCT with OPMD, *n* = 19). Exfoliative cytology samples were collected from buccal mucosa and OPMD lesions. MNs were manually counted to calculate RFMN. Nonparametric tests were applied to compare groups and the Wilcoxon signed‐rank test for intraindividual comparisons. Spearman's correlation and linear regression were used to evaluate associations with age and time.

**Results:**

RFMN in OPMD lesions (Group 3) was significantly higher than in the integrous mucosa of the same patients (median 8 MN/1000 cells, IQR 4–13; ~3.7‐fold increase; *p* = 0.001). No significant differences were observed between Groups 1 and 2 in integrous mucosa. RFMN in integrous mucosa positively correlated with time post‐HSCT (*ρ* = 0.694, *p* < 0.001), independent of age and GVHD presence.

**Conclusion:**

FA patients, particularly post‐HSCT with OPMD, exhibited markedly increased genomic instability. RFMN increases over time, including time post‐HSCT, emphasizing the need for vigilant long‐term oral monitoring in this high‐risk population.

## 1 Introduction

Patients with Fanconi anemia (FA), a rare autosomal recessive genetic disorder, have a higher predisposition to oral cancer, particularly oral squamous cell carcinoma (OSCC) [1, 2, 3]. One consequence of FA is bone marrow aplasia, which is most effectively treated with hematopoietic stem cell transplantation (HSCT) [1, 4, 5]. Although HSCT can correct bone marrow failure, it also increases the risk of malignant neoplasms in FA patients [2, 3, 4, 5, 6].

Exfoliative cytology has gained interest due to its simplicity and cost‐effectiveness in monitoring oral potentially malignant disorders (OPMD) [7, 8]. It is a useful method for following up on patients predisposed to OSCC, reducing the need for repeated biopsies, and is especially beneficial for those at higher risk of bleeding and infections, such as FA patients [1, 2, 3, 4, 5, 6]. However, the technique may miss early‐stage malignant cells in the superficial epithelium, requiring analysis of deeper mucosal layers for accurate results [9].

Micronuclei (MNs), which are chromosomal fragments or whole chromosomes lost during cell division, indicate chromosomal instability and can result from both spontaneous chromosomal changes and exposure to genotoxic substances such as alcohol and tobacco [10, 11, 12, 13, 14, 15, 16, 17, 18]. In FA patients, MNs may reflect defective DNA repair, a hallmark of the disease [1, 2, 3, 4, 5, 6]. Cellular biomarkers, such as the MN test, can improve accuracy and support the early assessment of potential malignant transformation in cells. The use of MNs for early detection of OSCC or OPMD in high‐risk populations, such as those with FA, may improve diagnosis before clinical symptoms appear [8, 10, 11, 12, 13, 14, 15, 16, 17, 18, 19].

Therefore, the present study aimed to compare the relative frequency of micronuclei (RFMN) in patients with FA in intact mucosa and in the presence of OPMD, and to evaluate the correlation of RFMN with time post‐HSCT.

## 2 Materials and Methods

### 2.1 Ethical Approval and Study Design

The Institutional Review Board of the Complexo Hospital de Clínicas, Universidade Federal do Paraná, approved the study for human research under protocol number CAAE: 53235921.3.0000.0096.

### 2.2 Participants

The study was conducted at a public hospital in southern Brazil specializing in the treatment of FA and participants were recruited in the FA outpatient clinic. Inclusion criteria were a confirmed diagnosis of FA, availability and provision of informed consent to participate in the study, and belonging to one of the following clinical groups:

1. FA without HSCT (Group 1).
2. FA post‐HSCT (Group 2).
3. FA post‐HSCT with OPMD (Group 3).

Exclusion criteria included medical conditions preventing sample collection, a previous diagnosis of OSCC, the presence of suspicious oral lesions under investigation, use of tobacco (smoked and smokeless forms) and alcohol or drug use disorder. Due to the rarity of the disease, all eligible patients who agreed to participate were included. Group allocation was based on clinical status (transplantation and presence of OPMD) to allow for comparative analyses. The participants in Group 3 were not receiving any treatment for OPMDs; at the time of the study, they were only being monitored.

The sample size calculation was based on the analysis of MN differences between intact mucosa and OPMD in Group 3, using the lowest effect size reported in previous meta‐analyses as reference (SMD = 1.35) [20]. For a paired test, with *α* = 0.05 and 80% power, the minimum required number of patients was seven pairs. As 19 patients were included in Group 3, the study had sufficient power to detect significant differences in this comparison. Comparisons involving Groups 1 and 2 were conducted exploratorily. The final total sample included 46 patients.

### 2.3 Sample Collection

Exfoliative cytology of epithelial cells was performed on the lateral borders of the tongue and bilaterally on the buccal mucosa of all participants, regardless of whether they presented OPMD or intact mucosa, as well as on mucosae with clinically identified OPMD.

The clinically identified OPMDs included leukoplakia, erythroplakia, actinic cheilitis, and oral lichenoid lesions [21]. Incisional biopsies were performed only when necessary for monitoring or treatment purposes; they were not included in the study and were not conducted solely to confirm clinical diagnosis.

Epithelial cells were collected using an endocervical brush (cytobrush), spread in duplicate on glass slides, and fixed in absolute alcohol for 2 h [9, 22]. Staining was performed using the Feulgen reaction, with samples immersed in HCl and Schiff reagent to stain DNA pink/purple. Cells were then dehydrated in ethanol and cleared in xylene [10].

The analysis was performed using an optical microscope. The total cell count was estimated at ×100 magnification. Visualization, identification, and counting of MN were performed at ×400 magnification, following the basic criteria proposed by Tolbert et al. [10]. A micronucleated cell should exhibit intact and relatively flat cytoplasm; minimal or no overlap with adjacent cells; minimal debris; and a normal, intact nucleus with a smooth and distinct nuclear perimeter.

A MN was included if it had a rounded, smooth perimeter suggestive of a membrane; was less than one‐third the diameter of the associated nucleus yet large enough to discern its shape and color; was Feulgen positive; had staining intensity and texture similar to that of the nucleus; was in the same focal plane as the nucleus; and showed no overlap or connection with the nucleus (Figure 1) [10].

**FIGURE 1:**
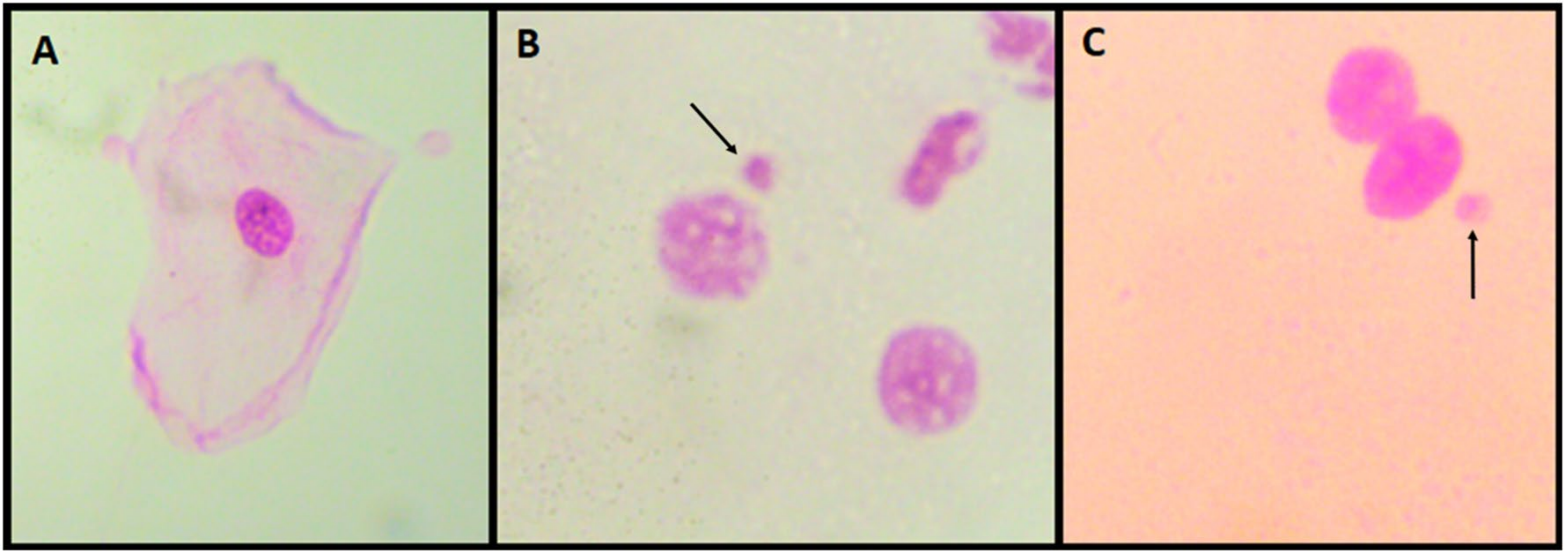
Exfoliated buccal epithelial cells from patients with FA stained with the Feulgen reaction (×400 magnification). (A) A normal cell with a single, intact nucleus can be observed. (B, C) Micronuclei (arrows) are identified, indicating chromosomal instability.

### 2.4 Calculation of the RFMN

The MN frequency was determined by manually counting the total number of cells, micronucleated cells, and the total number of MNs observed. The number of MNs per patient was RFMN, as described by Ramírez et al. [13].

MN identification and counting were performed by a single examiner following standardized criteria. All samples were analyzed under the same conditions to ensure consistency in MN identification. The examiner was previously trained in MN identification based on established morphology.

### 2.5 Statistical Analysis

Descriptive statistics were calculated for demographic, clinical, and RFMN data. Continuous variables were reported as mean ± standard deviation (SD) if normally distributed, or as median (*Q*1–*Q*3) if non‐normally distributed. Categorical variables were presented as absolute counts and percentages. Comparisons between independent groups (Groups 1, 2, and 3) were performed using Mann–Whitney *U* or Kruskal–Wallis tests, according to the number of groups and data distribution. Intraindividual comparisons of RFMN between integrous mucosa and OPMD lesions (Group 3) were conducted using the Wilcoxon signed‐rank test. Correlations between continuous variables (e.g., RFMN in integrous mucosa and time post‐HSCT) were assessed using Spearman's rank correlation. Linear regression was applied to adjust for potential confounders when appropriate. A significance level of *p* < 0.05 was adopted for all analyses. Statistical analyses were performed using IBM SPSS Version 20.

## 3 Results

The sample comprised 46 patients with FA, divided into three groups: Group 1 (FA without HSCT, *n* = 14), Group 2 (FA post‐HSCT without OPMD, *n* = 13), and Group 3 (FA post‐HSCT with OPMD, *n* = 19). Demographic and clinical characteristics of the participants are presented in Table 1.

**TABLE 1:** Sample characteristics (*n* = 46).

	Group 1, *n* = 14	Group 2, *n* = 13	Group 3, *n* = 19	Total^a^
Age	14.2 ± 6.5	17.1 ± 7.1	24.4 ± 8.4	20.9 ± 8.6
Gender				
Female	8 (57.1)	6 (46.2)	13 (68.4)	27 (58.7)
Male	6 (42.9)	7 (53.8)	6 (31.6)	19 (41.3)
Race				
White	2 (14.3)	5 (38.5)	8 (42.1)	15 (32.6)
Non‐White	12 (85.7)	8 (61.5)	11 (57.9)	31 (67.4)
HSCT type				
Haploidentical	—	3 (23.1)	5 (26.3)	8 (25)
Related	—	7 (53.8)	8 (42.1)	15 (46.9)
Unrelated	—	3 (23.1)	6 (31.6)	9 (28.1)
Donor				
Mother	—	1 (7.7)	2 (10.5)	3 (9.4)
Father	—	0	1 (5.3)	1 (3.1)
Sibling	—	7 (53.8)	8 (42.1)	15 (46.9)
Uncle	—	0	2 (10.5)	2 (6.2)
Cousin	—	2 (15.4)	0	2 (6.2)
Unrelated	—	3 (23.1)	6 (31.6)	9 (28.1)
Years post‐HSCT	—	7.7 ± 5.2	11 (4–18)^b^	9.5 (6–13)^b^
GVHD history				
Yes	—	2 (15.4)	8 (42.1)	10 (31.2)
No	—	11 (84.6)	11 (57.9)	22 (68.8)
OPMD				
Actinic cheilitis	—	—	2 (10.5)	2 (10.5)
OLL	—	—.	6 (31.6)	6 (31.6)
Leukoplakia	—	—	8 (42.1)	8 (42.1)
Erythroleukoplakia	—	—	3 (15.8)	3 (15.8)
Total	14 (100)	13 (100)	19 (100)	46 (100)

*Note:* Group 1: FA without HSCT; Group 2: FA post‐HSCT without OPMD; and Group 3: FA post‐HSCT with OPMD.

Abbreviations: FA: Fanconi anemia; GVHD: graft‐versus‐host disease; HSCT: hematopoietic stem cell transplantation; OLL: oral lichenoid lesions; OPMD: oral potentially malignant disorders.

^a^ Total calculated considering only the groups that presented the variable.

^b^ Non‐normal distributed variables are reported by median (*Q*1–*Q*2).

The RFMN in integrous mucosa showed a non‐normal distribution (*p* = 0.003). Comparisons between independent groups (Group 1 vs. Group 2 and Group 3) did not reveal statistically significant differences in RFMN in integrous mucosa (*p* > 0.2).

Within Group 3, the RFMN in OPMD lesions was significantly higher than in the integrous mucosa of the same individuals (Wilcoxon signed‐rank test, *p* = 0.001). Sixteen out of 17 patients showed an increase. The MN frequency in lesions was approximately 3.7 times higher than in integrous mucosa (95% CI: 2.2–6.1), with a median fold increase of 4.0. Descriptive values for RFMN in integrous mucosa, OPMD lesions, and their difference in Group 3 are summarized in Table 2.

**TABLE 2:** RFMN in patients with FA post‐HSCT in integrous mucosa and OPMD.

	Group 1	Group 2	Group 3	Total
RFMN in integrous mucosa	1.7/1000 ± 1.5/1000	2.3/1000 ± 1.7/1000	2/1000 (1/1000–3.5/1000)^a^	2/1000 (1/1000–3/1000)^a^
RFMN in OPMD	—	—	8/1000 (4/1000–13/1000)^a^	—
Difference	—	—	6/1000 (2/1000–10/1000)^a^	—

*Note:* Group 1: FA without HSCT; Group 2: FA post‐HSCT without OPMD; and Group 3: FA post‐HSCT with OPMD.

Abbreviations: FA: Fanconi anemia; HSCT: hematopoietic stem cell transplantation; OPMD: oral potentially malignant disorders; RFMN: relative frequency of micronuclei.

^a^ Non‐normally distributed variables are reported by median (*Q*1–*Q*2).

Spearman's correlation analyses, performed due to the nonnormal distribution of all RFMN variables, are presented in Table 3. The strong and significant correlation between RFMN in integrous mucosa and time post‐HSCT in the combined Groups 2 and 3 remained significant after adjustment for age and GVHD presence (*β* = 0.691, *p* < 0.001).

**TABLE 3:** Spearman's correlations between RFMN and time variables in FA patients.

	RFMN in integrous mucosa	RFMN in OPMD	Total RFMN
Age	0.0342 (*p* = 0.023)* [*n* = 44]	0.06 (*p* = 0.808) [*n* = 19]	0.0582 (*p* < 0.001)** [*n* = 34]
Age at HSCT	–0.156 (*p* = 0.411) [*n* = 30]	0.03 (*p* = 0.904) [*n* = 19]	0.158 (*p* = 0.387) [*n* = 32]
Years post‐HSCT	0.694 (*p* > 0.001)** [*n* = 30]	0.094 (*p* = 0.701) [*n* = 19]	0.535 (*p* = 0.002)** [*n* = 32]

Abbreviations: FA: Fanconi anemia; HSCT: hematopoietic stem cell transplantation; OPMD: oral potentially malignant disorders; RFMN: relative frequency of micronuclei.

* Correlations significant at 0.05 level.

** Correlations significant at 0.001 level.

Exploratory analyses did not reveal significant associations between RFMN and other demographic variables, transplant type, donor, or lesion type. Trends were observed for higher RFMN in integrous mucosa in females (*p* = 0.081) and in lesions according to HSCT donor type (*p* = 0.069).

## 4 Discussion

This study investigated genomic instability in the buccal mucosa of patients with FA, comparing RFMN in integrous mucosa and OPMD lesions, and evaluating its association with time post‐HSCT. Our results demonstrate that RFMN is significantly elevated in OPMD lesions compared with integrous mucosa within the same patients, with a median 3.7‐fold increase. In contrast, no statistically significant differences were observed between FA patients without HSCT and post‐HSCT patients without OPMD, highlighting the importance of lesion‐specific cytogenetic changes. Additionally, a strong positive correlation was found between RFMN in integrous mucosa and time post‐HSCT, suggesting cumulative genomic instability over time. Trends observed for sex and HSCT donor type did not reach significance, likely due to limited sample size. Collectively, these findings underscore that FA patients, particularly those post‐HSCT with OPMD, exhibit pronounced chromosomal instability and may benefit from targeted long‐term oral surveillance.

To date, no comparable studies have reported MN frequencies specifically in OPMD lesions of FA patients. In our cohort, the overall median RFMN in OPMD lesions post‐HSCT was 8 MN/1000 cells (interquartile range 4–13), substantially higher than the RFMN reported in non‐FA OPMD populations by Gupta et al., who found mean values of 2.3 MN/1000 in oral leukoplakia, 1.5 MN/1000 in oral submucous fibrosis, and 1.7 MN/1000 in oral lichen planus [23]. Although direct comparisons should be interpreted with caution due to differences in patient populations and measurement methods, these results suggest that FA patients post‐HSCT exhibit markedly increased genomic instability in OPMD lesions.

Only one study in the literature, conducted by Ramírez et al., has applied the MN frequency test to buccal epithelial cells of patients with FA [13]. Their study reported that patients with FA exhibit higher RFMN in integrous buccal mucosa than healthy controls. In our study, we compared RFMN between integrous mucosa and OPMD lesions within the same FA patients and found that RFMN in OPMD lesions was approximately 3.7 times higher than in integrous mucosa. Notably, there appears to be a trend of increasing RFMN from healthy subjects to FA patients' integrous mucosa, and further to OPMD lesions in FA patients. This suggests that RFMN is more pronounced in oral tissues with higher cancer risk, even though a clear progression pathway cannot be established.

Patients with OPMD remain at elevated risk for OSCC development, which may arise either from the lesion itself or from clinically normal‐appearing mucosa, as areas that appear unaffected may still harbor cytological and molecular alterations [12, 21, 23, 24, 25, 26, 27].

The expected MN frequency for the general population (non‐FA patients) ranges from 0.5 to 2.5 MN/1000 cells. Therefore, patients with an MN frequency above 2.5 are considered at higher risk of developing OSCC [13, 15, 18]. In our study, the median RFMN in integrous mucosa of FA patients corresponded to approximately 2 MN/1000 cells, which falls within the normal range, whereas the median RFMN in OPMD lesions was approximately 8 MN/1000 cells, exceeding the upper limit and suggesting an increased risk for OSCC. When comparing the RFMN to the reference range for the general population (0.5–2.5 MN/1000 cells), approximately 25% of FA patients had RFMN above this threshold in integrous mucosa, while all OPMD lesions exceeded this limit. This highlights a marked increase in genomic instability in OPMD compared with integrous mucosa within the same patients.

In our study, FA patients without HSCT (Group 1) and post‐HSCT patients without OPMD (Group 2) exhibited median RFMN values of 1.7 and 2.3 MN/1000 cells, respectively. These findings appear to contrast with Ramírez et al., who reported higher MN frequencies in FA patients without HSCT. This discrepancy may be explained by differences in the analysis units (independent cheek observations vs. per‐patient assessment), sample size, and variability between groups. Our findings align with literature indicating that HSCT increases the predisposition to OSCC in FA patients [1, 2, 4, 6]. Although the hematologic disorder is treated, genetic alterations in FA patients persist, independently elevating the risk of malignancies, and there is an added risk due to the combined effects of HSCT and FA predisposition to malignancies, particularly those of epithelial origin [19].

In our cohort, OPMD lesions were observed exclusively in FA patients post‐HSCT; no cases were identified in patients without HSCT. This finding is consistent with the literature on long‐term posttransplant complications. US cohort studies have reported a cumulative incidence of posttransplant solid malignancies of 5.6% at 13 years and 6.1% at 10 years, highlighting the persistent risk of secondary cancers among HSCT survivors [24, 28]. These data underscore the importance of careful oral surveillance in this high‐risk population.

As expected, a moderate correlation was observed between age and total RFMN, as well as RFMN in integrous mucosa. In addition, we observed a strong positive correlation between time post‐HSCT and RFMN in integrous mucosa (Spearman's *ρ* = 0.694, *p* < 0.001), which remained significant after adjusting for age and GVHD presence (*β* = 0.691, *p* < 0.001). This suggests that genomic instability may accumulate over time following HSCT, independent of patient age or GVHD. Taken together, these findings reinforce that clinical surveillance appears to be more strongly driven by age and the presence of OPMD and emphasize the importance of long‐term oral surveillance in HSCT survivors, as both the presence of OPMD and the progressive increase in MN frequency may contribute to elevated cancer risk. The lack of correlation between RFMN in OPMD may be explained by the smaller sample size, as this analysis was restricted to Group 3.

The diagnostic performance reported for MN analysis, together with the findings of the present study, suggests potential clinical applicability as an adjunctive tool for the surveillance and follow‐up frequency consideration in FA. Nevertheless, MN analysis should not be considered a substitute for careful oral examination, histopathological evaluation of suspicious lesions, or established surveillance protocols. Future longitudinal studies are warranted to establish clinically relevant thresholds and determine whether serial MN assessment can predict malignant transformation or improve early detection of OSCC in patients with FA.

The authors acknowledge several limitations of this study, including the absence of a control group, the strictly clinical diagnosis of OPMD, and the manual scoring of MNs, as no automated software was used. Intraobserver reliability was not formally assessed, which may represent a limitation of this study. However, all analyses were performed by a single examiner using standardized and widely accepted criteria, which may have reduced variability. Additionally, the sample size may be considered small for some analyses, providing sufficient power only to detect strong correlations. Consequently, trends such as higher RFMN in integrous mucosa in females or variations in lesions according to HSCT donor type may not have reached statistical significance due to limited sample size. Further studies with larger and more representative cohorts are needed to address these limitations.

## 5 Conclusion

In FA patients, MN frequency is markedly higher in OPMD lesions than in integrous mucosa, reflecting increased genomic instability associated with higher oral cancer risk. RFMN in integrous mucosa also increases with time post‐HSCT, indicating a cumulative effect of transplantation on genomic stability. These findings emphasize that RFMN has clinical applicability in oral monitoring FA survivors.

## Author Contributions

**Bárbara Soldatelli Ballardin:** conceptualization, data curation, investigation, methodology, validation, visualization, writing – original draft preparation, review and editing. **Pedro Leonardo Czmola de Lima:** data curation, statistical analyses, writing – reviewed draft preparation and editing. **Pâmela Olivia de Moura:** data curation, investigation, validation, visualization, writing – original draft preparation, review and editing. **Juliana Lucena Schussel:** conceptualization, formal analysis, investigation, methodology, project administration, supervision, validation, visualization, writing – original draft preparation, review and editing. **Cassius Carvalho Torres‐Pereira:** conceptualization, formal analysis, investigation, methodology, project administration, supervision, validation, visualization, writing – original draft preparation, review and editing.

## Funding

This study was financed in part by the Coordenação de Aperfeiçoamento de Pessoal de Nível Superior – Brasil (CAPES) – Finance Code 001, Brazil.

## Conflicts of Interest

The authors declare no conflicts of interest.

## Data Availability

The data that support the findings of this study are available from the corresponding author upon reasonable request.
